# SARS-CoV-2 neutralizing antibodies in patients with varying severity of acute COVID-19 illness

**DOI:** 10.1038/s41598-021-81629-2

**Published:** 2021-01-21

**Authors:** Chandima Jeewandara, Deshni Jayathilaka, Laksiri Gomes, Ananda Wijewickrama, Eranga Narangoda, Damayanthi Idampitiya, Dinuka Guruge, Ruwan Wijayamuni, Suranga Manilgama, Graham S. Ogg, Chee Wah Tan, Lin-Fa Wang, Gathsaurie Neelika Malavige

**Affiliations:** 1 Centre for Dengue Research, Faculty of Medical Sciences, University of Sri Jayawardenapura, Nugegoda, Sri Lanka; 2 National Institute of Infectious Diseases, Angoda, Sri Lanka; 3 Allergy, Immunology and Cell Biology Unit, University of Sri Jayewardenepura, Nugegoda, Sri Lanka; 4 Colombo Municipality Council, Colombo, Sri Lanka; 5 National Hospital Kandy, Kandy, Sri Lanka; 6 MRC Human Immunology Unit, MRC Weatherall Institute of Molecular Medicine, University of Oxford, Oxford, UK; 7 Programme in Emerging Infectious Diseases, Duke-NUS Medical School, Singapore, Singapore

**Keywords:** Adaptive immunity, Infection, Infectious diseases, Immunology, Microbiology, Medical research, Pathogenesis

## Abstract

In order to support vaccine development, and to aid convalescent plasma therapy, it would be important to understand the kinetics, timing and persistence of SARS-CoV-2 neutralizing antibodies (NAbs), and their association with clinical disease severity. Therefore, we used a surrogate viral neutralization test to evaluate their levels in patients with varying severity of illness, in those with prolonged shedding and those with mild/asymptomatic illness at various time points. Patients with severe or moderate COVID-19 illness had earlier appearance of NAbs at higher levels compared to those with mild or asymptomatic illness. Furthermore, those who had prolonged shedding of the virus, had NAbs appearing faster and at higher levels than those who cleared the virus earlier. During the first week of illness the NAb levels of those with mild illness was significantly less (p = 0.01), compared to those with moderate and severe illness. At the end of 4 weeks (28 days), although 89% had NAbs, 38/76 (50%) in those with > 90 days had a negative result for the presence of NAbs. The Ab levels significantly declined during convalescence (> 90 days since onset of illness), compared to 4 to 8 weeks since onset of illness. Our data show that high levels of NAbs during early illness associated with clinical disease severity and that these antibodies declined in 50% of individuals after 3 months since onset of illness.

## Introduction

The 2019 novel coronavirus (SARS-CoV-2) virus has resulted in over 36 million infections and one million deaths in a period of just 9 months^1^. While many countries are under various degrees of lockdown there is a huge race to develop a safe and effective vaccine, and currently 21 vaccine candidates undergoing clinical trials^2^. The main aim of vaccination is to induce long lasting protection against infection with the SARS-CoV-2 by inducing a robust virus specific neutralizing antibody (NAb) and T cell response. In addition, there are many clinical trials underway to assess the benefit of the use of convalescent plasma treatment of COVID-19 and use of monoclonal antibodies to block virus attachment^3,4^. Therefore, it is important to understand the evolution of the NAb responses in patients with varying severity of COVID-19 illness, their association with viral clearance, and to study the persistence of them in those who have been naturally infected with the SARS-CoV-2.

Convalescent plasma treatment of those with severe acute respiratory syndrome coronavirus (SARS-CoV) and Middle East respiratory syndrome coronavirus (MERS-CoV) was shown to reduce mortality, duration of hospital stay, clinical improvement and viral loads, which have been attributed to the presence of the neutralizing antibodies (NAbs)^5,6^. Convalescent plasma has been used to treat many patients with severe COVID-19 illness, which has been safe and reported to reduce mortality, although no data are available from randomized, clinical trials^7,8^. Analysis of SARS-CoV-2 specific NAbs from infected individuals showed that the majority of such antibodies target the receptor binding domain (RBD) and prevent binding to the host cell receptor ACE2^9^. However, there have been recent concerns regarding decline of both total antibodies and NAbs to SARS-CoV-2 at 8 weeks since onset of illness, especially in those with mild illness^10^. This decline in antibody titres was seen more for the total IgG antibodies than the NAbs, suggesting that NAbs that are needed for subsequent protection could be long lasting. Following MERS-CoV and SARS-CoV infection, functional NAbs were shown to persist for over 1 year^11^ and up to at least 17 years^12^, although they were undetectable in significant proportion of individuals by 30 to 34 months^13,14^. Antibody responses to other seasonal coronaviruses also have shown to be short-lived and that individuals could be infected with coronaviruses such as NL63 within a 6 month period^15^. Therefore, in order to develop effective vaccines, it would be important to answer key questions such as if the appearance of NAbs led to less severe disease, stopped virus shedding and their persistence. In this study, we initially investigated the kinetics of SARS-CoV-2 specific NAbs in a cohort of patients with varying severity of illness, then proceeded to further characterize the responses at different time points in relation to clinical disease severity and also investigate their persistence of NAbs after 90 days since onset of illness.

## Results

### Determining specificity of the sVNT in measuring SARS-CoV-2 NAbs in the Sri Lankan population

In order to determine the specificity of the sVNT in Sri Lankan individuals, we initially assessed the % of inhibition in 81 serum samples obtained from individuals who presented with a febrile illness to the outpatient department of the National Institute of Infectious Diseases (NIID), Sri Lanka in 2018. All these individuals had a % of inhibition less than the cut of value of 25%. We then assessed the specificity of the assay in 285 non-exposed individuals recruited from the Colombo Municipality area during the month of April. The percentage of inhibition in this population was also less than the cut-off value. Therefore, the specificity of this assay was found to be 100% as previously described^16^.

### Longitudinal changes in NAbs in patients with varying severity of clinical disease

**Figure 1:**
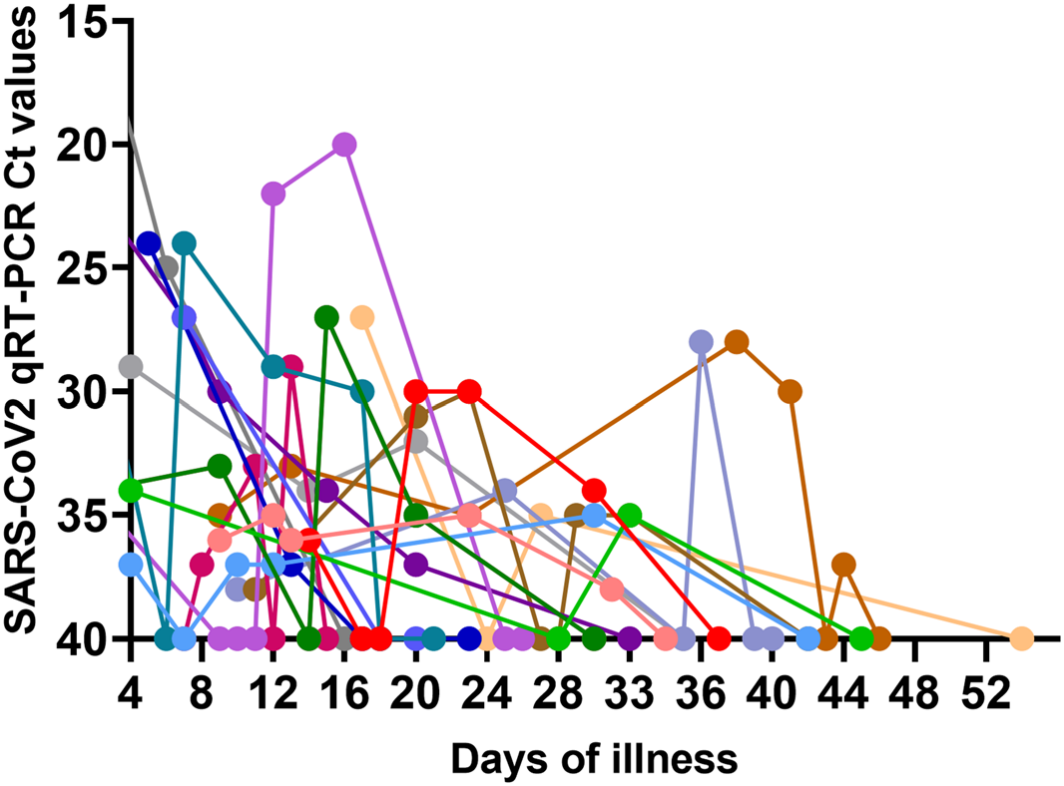
Patterns of SARS-CoV-2 viral shedding in respiratory samples in patients with prolonged COVID-19 illness. Realtime qPCR was carried out in individuals with prolonged shedding (n = 17) throughout the course of illness to determine the patterns and duration of virus shedding.

In order to determine the longitudinal changes of SARS-CoV-2 NAbs in patients with varying clinical disease severity, we assessed their levels in those with severe (n = 6), moderate (n = 5) and mild/asymptomatic illness (n = 13) and also those who had mild illness but with prolonged shedding of the virus (n = 21). Those who shed the virus for over 25 days were considered as those with prolonged shedding. Of the 21 individuals with prolonged shedding, there were 10 individuals who had shedding for over 50 days. The patterns of virus shedding in those with mild but prolonged illness is shown in Fig. 1.

In patients with severe and moderate illness and in those with prolonged shedding, blood samples were obtained during the first week, second week and at the time when they were discharged from the hospital (4 to 6 weeks since onset illness). As those with mild illness were discharged from hospital during the second week of illness, the first two blood samples (1st week and 2nd week) were obtained while in hospital and again their third blood sample was obtained after discharging from the hospital, while they were at home (4 to 6 weeks since onset of illness).

**Figure 2:**
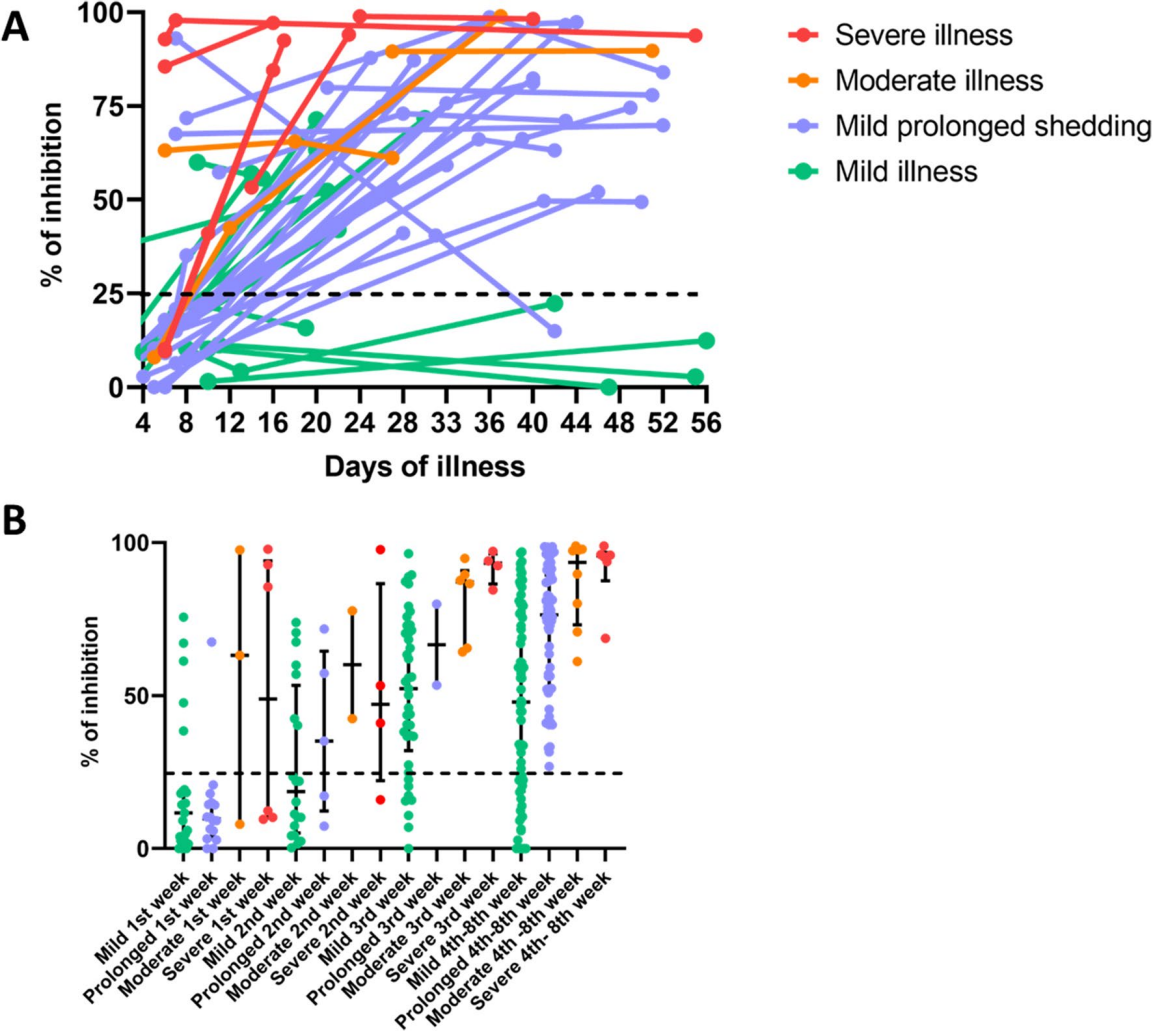
Longitudinal analysis SARS-CoV-2 neutralizing antibodies in patients with varying severity of COVID-19. SARS-CoV-2 NAbs were measured in those with severe (n = 6), moderate (n = 5) and mild/asymptomatic illness (n = 13) and also those who had mild illness but with prolonged shedding of the virus (n = 21) (**A**). In order to assess the timing of the NAb responses at different time point, these antibodies were measured in patients with severe pneumonia (n = 10), moderate illness (n = 19), mild illness (n = 150) and prolonged shedding (n = 82). The NAbs levels were determined in this larger cohort of individuals during the first 3 weeks and during week 4 to 8 of illness (**B**). The black dotted line indicates the cut-off value of a positive result.

In the longitudinal analysis of NAbs, they appeared earlier and faster, at higher levels in those who had severe and moderate pneumonia, followed by those who had prolonged shedding, while they appeared later, at lower levels in those who had mild/asymptomatic disease (Fig. 2A). 4/13 individuals with mild illness had no detectable NAbs even 40 days since onset of illness, whereas only 1/21 individuals with prolonged shedding had NAbs below detection level (< 25% of inhibition).

### The timing of the appearance of NAbs and their levels in relation to clinical disease severity

In order to further evaluate the appearance and the quantity of NAbs in relation to clinical disease severity, we assessed antibodies in blood samples at different time points from patients with severe pneumonia (n = 10), moderate illness (n = 19), mild illness (n = 150) and prolonged shedding (n = 82). The NAbs levels were determined in this larger cohort of individuals during the first 3 weeks and during week 4 to 8 of illness.

Again, those with moderate and severe illness had higher NAbs levels (median 63.16 and 48.9% of inhibition) during the 1st week, and in all subsequent time points compared to those with mild and prolonged shedding (Fig. 2A). During the first week of illness the NAb levels of those with mild/prolonged illness (median = 10.3, IQR 3.5 to 18.2% of inhibition) was significantly less (p = 0.01), compared to those with moderate and severe illness (median 63.2, IQR 9.9 to 95.2% of inhibition). There was no difference in the NAb levels during the first week of illness in those with mild illness and mild but prolonged illness (p = 0.71). After the 3rd week (4th to 8th week), although all patients with severe, moderate and prolonged shedding had a positive test result, 23/69 (33.3%) of those with mild/asymptomatic illness were negative (% of inhibition < 25). Those with prolonged shedding, who had mild or asymptomatic illness had significantly higher (p < 0.0001) NAb levels (median 76.4%, IQR 52.32 to 89.5% of inhibition) than those with mild/asymptomatic illness (median 47.9%, IQR 18.9 to 77.1% of inhibition) during week 4 to 8. This data further reinforces the longitudinal analysis of NAbs as shown in Fig. 2B.

### SARS-CoV-2 NAb positivity at different time points and persistence

**Figure 3:**
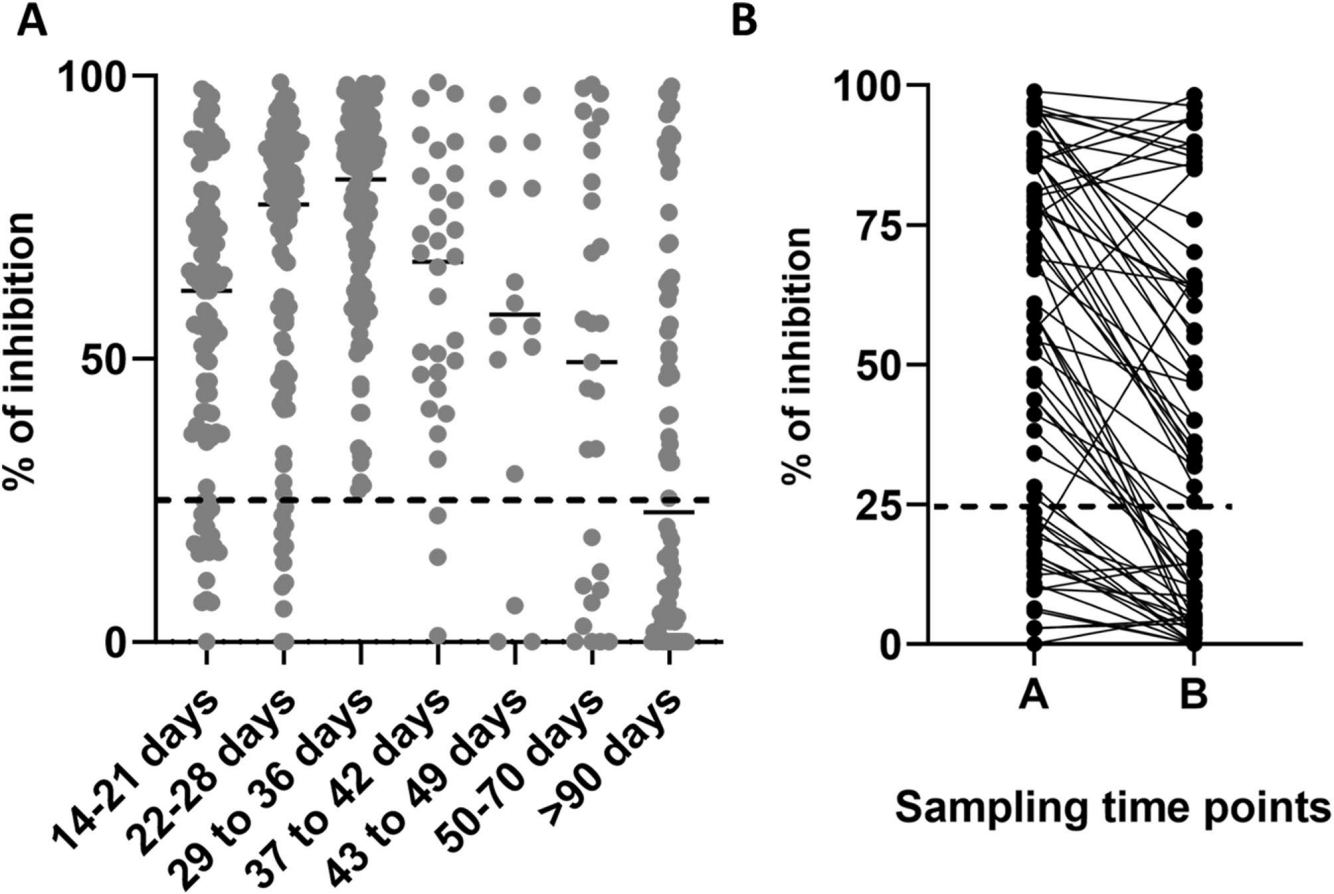
SARS-CoV-2 neutralizing antibody positivity during different weeks since onset of illness. SARS-CoV-2 NAbs were measured at different time points from patients with severe pneumonia (n = 10), moderate illness (n = 19), mild illness (n = 150) and prolonged shedding (n = 82) (**A**) and also measured at different time points irrespective of disease severity. 14 to 21 (n = 96), day 22 to 28 (n = 99), day 29 to 36 (n = 132), day 37 to 42 (n = 32), day 43 to 49 (n = 16), between ay 50 to 70 (n = 29) and more than 90 day (n = 76) (**B**), Responses were also measure during early illness (4 to 8 weeks, time point A) and during convalescence (> 90 days since onset of illness, time point B). The black dotted line indicates the cut-off value of a positive result.

We then proceeded to assess the detection of NAbs at various time points in illness, irrespective of clinical disease severity and also to assess if NAbs persisted over 90 days since onset of illness. NAbs were measured by the sVTN on day 14 to 21 (n = 98), day 22 to 28 (n = 100), day 29 to 36 (n = 132), day 37 to 42 (n = 32), day 43 to 49 (n = 16), day 50 to 70 (n = 29) and > 90 days (n = 76). The positivity rates during day 14 to 21 was 79.8%, day 22 to 28 was 89%, day 29 to 36 was 100%. Therefore, all patients tested positive by the end of 5th week of illness (Fig. 3A). At the end of 4 weeks (28 days), although 89% had NAbs of a sufficient quantity (positive result), 11/89 (12.3%) of these individuals had NAbs titres < 50%. The NAb positivity rates declined after 5 weeks as the positivity was 90.6% at 37 to 42 days, 65.5% between 50 to 70 days of illness, and 38/76 (50%) in those with > 90 days since onset of illness suggesting that the NAbs could be declining with time. The NAb titres in the majority of individuals were significantly higher (p < 0.0001) in the blood samples taken during early illness (time point A, median 56.3, IQR 16.7 to 84.4% of inhibition) compared to the samples taken after 90 days since onset of illness (time point B, median 22.3, IQR 3.7 to 63,3% of inhibition) (Fig. 3B). However, in 8/76 (10.5%) individuals, the NAb titres rose from time point A to B. Of the NAb that were assessed in the 76 individuals > 90 days since onset of illness, the NAb titres were > 85% in the 3 individuals who had severe illness and was 75.6% in those who had moderate pneumonia. All other 72 patients had mild or asymptomatic illness.

## Discussion

In this study, we show that the early appearance of SARS-CoV-2 NAbs at high levels was not associated with milder disease nor with early clearance of the virus. Early appearance of NAbs has previously shown to occur in those with severe disease compared to those with mild illness^17^. It was recently shown that a high frequency of extrafollicular B cells development is seen in COVID-19, which correlated with disease severity. These extrafollicular B cells were responsible for development of development of SARS-CoV-2 specific neutralizing antibodies, very early during illness, which was shown to associate with severe disease^18^. In addition to production of NAbs by extrafollicular B cells, the early appearance of NAbs in patients with more severe disease could be due to the boosting of NAbs specific to previous coronaviruses. Therefore, early appearance of such cross-reactive antibody responses could have a potential to cause severe illness by antibody dependent enhancement^19^. Higher initial viral loads were associated with progression to more severe disease in SARS^20,21^. Therefore, higher viral loads could drive a more robust NAb response. However, infants who were symptomatic had higher nasopharyngeal viral loads, but less severe illness compared to older children with more severe illness^22^, suggesting that higher viral loads were not necessarily associated with more severe illness.

It was seen that by the end of 4 weeks, 11% of individuals did not have adequate quantities of NAbs. Since the onset of the outbreak, due to the lack of specific treatment options, convalescent plasma of recovered patients with COVID-19 has been used to treat patients with moderate or severe COVID-19 illness^23,24^. The FDA, USA has issued an emergency use authorization for the use of convalescent plasma as it was believed that it may be effective^23^. Although randomized clinical trials have not been conducted to determine the efficacy of this treatment, as use of convalescent plasma depends on the presence of high levels of NAbs, it is recommended to test the presence of high titres, before selection of potential donors^25,26^.

The relationship between the appearance of NAb with duration of virus shedding has not been previously studied. Surprisingly, those who had prolonged shedding had higher levels of NAbs than those who cleared the virus, and the NAbs appeared in such prolonged shedders earlier than in those who cleared the virus earlier. In Sri Lanka, until recently, patients with COVID-19 were only discharged from hospital if they had 2 negative PCRs, 24 h apart. Therefore, despite these prolonged shedders developing antibodies earlier than those who cleared the virus, and at higher titres, they still continued to shed the virus. Although the majority of such prolonged shedders had lower viral titires (Ct values > 30), some individuals still had higher viral loads even after 30 days of illness. As many other countries do not keep patients in hospital until they become PCR negative, the relationship between early appearance of NAbs and yet persistence has not been documented previously and questions the role of NAb alone in viral clearance.

Although NAbs are thought to associate with protection, this has not been the case with infections such as dengue, which induce cross reactive antibodies as seen between different coronaviruses. Those with high NAbs for a particular dengue virus serotype were found to get re-infected with the same serotype^27^. In addition, the kinetics of NAbs levels in those with varying severity of dengue, was remarkably different based on the infecting dengue virus serotype^28^. Therefore, it is crucial to carry out further studies to identify the protective antibody responses for the SARS-CoV-2, their persistence and their ability to prevent re-infection. As it is clearly evident that patients with COVID-19 had varying levels of NAbs at recovery, it would be important to assess their NAbs by using a simple assay such as this sVNT, for selection of suitable donors.

Although the majority of individuals appeared to be antibody positive by end of week 5, the positivity rates declined thereafter. The decline in NAb antibodies in COVID-19 patients has been documented in recent reports^17^, which has implications in providing long lasting immunity through vaccination. Although we only tested NAbs in a small cohort of individuals with more 90 days since onset of illness, none of those with mild/asymptomatic illness had NAb above the cut-off value. Further studies are required to determine if such individuals have memory B cell responses and functional Nab even at low levels that would prevent re-infection.

In summary, we show that the early appearance of SARS-CoV-2 NAbs at high levels was not associated with milder disease nor with early clearance of the virus and that NAbs did not persist in those with mild/asymptomatic illness.

## Methods

### Patients

Patients were recruited from the National Institute of Infectious Diseases (NIID), Sri Lanka and the Theldeniya Covid-19 Management Centre in Kandy. The patients were followed throughout their illness while they were in hospital and the severity grading was based on the worst severity while in hospital. Clinical disease severity was classified as mild, moderate and severe according to the WHO guidance of COVID-19 disease severity^29^. Accordingly, those who had a confirmed symptomatic SARS-CoV-2 infection with either fever, cough, fatigue, anorexia, myalgia and shortness of breath and with non-specific symptoms such as sore throat, headache and diarrhoea, but with no evidence of hypoxia or pneumonia were classified as having mild illness. Those with clinical signs of pneumonia with a respiratory rate of > 30 breaths/min, or with SpO_2_ < 90% on room air were considered as having severe pneumonia^29^. Those with clinical and radiological signs of pneumonia, but who did not fulfill the criteria of severe disease were classified as having moderate illness. The clinical data of all patients were retrieved from their clinical records within one week after discharge from hospital and entered to case record forms. Their clinical records contained all clinical details including clinical features, radiological investigation details, laboratory investigations and their results on different days. Although many patients with mild illness recovered from illness during the first week since onset of symptoms, they had to be hospitalized until they had two negative PCRs, 24 h apart. Until early July 2020, COVID-19 patients in Sri Lanka were only discharged from hospital when they had two negative PCRs, 24 h apart, which led to many patients being hospitalized for prolonged periods due to continued shedding of the virus^30^. Therefore, for the purpose of the evolution of the antibody response, we assessed presence of Nabs since onset of illness, rather than correlating responses with the duration of illness.

### Recruitment of patients for longitudinal analysis of Nabs in relation to clinical disease severity

In order to determine the changes of Nabs with varying clinical disease severity, we assessed their levels in those with severe (n = 6), moderate (n = 5) and mild/asymptomatic illness (n = 13) and also those who had mild illness but with prolonged shedding of the virus (n = 21). Blood samples were obtained during the first week, second week and when they were discharged from hospital (4 to 6 weeks since onset illness) in all patients except the two patients who succumbed to their illness during the end of the second week since onset of illness. The duration of illness was defined from the day or onset of symptoms and not the day or PCR positivity or admission to hospital.

The median duration of virus shedding in this whole cohort was 25 days (IQR 15 to 38 days) and therefore, those who had virus shedding for over 25 days and hospitalized for over 25 days were considered to have prolonged shedding of the virus.

### The timing of the appearance of NAbs and their levels in relation to clinical disease severity

Since only a limited number of patients were included in the longitudinal analysis of the NAbs in patients with varying severity of illness (n = 36), we recruited additional patients to determine the appearance of NAbs at different time points. For this purpose, we assessed antibodies in blood samples at different time points from patients with severe pneumonia (n = 10), moderate illness (n = 19), mild illness (n = 150) and prolonged shedding (n = 82). The NAbs levels were determined in this larger cohort of individuals during the first 3 weeks and during week 4 to 8 of illness. The duration of illness was defined from the day or onset of symptoms and not the day or PCR positivity or admission to hospital. In the patients who were completely asymptomatic, the day of illness was defined as the day of PCR positivity.

### SARS-CoV2 NAb positivity at different time points and persistence

Since the majority of the patients with COVID-19 who were admitted to NIID with acute illness were from the Colombo Municipality Council area, blood samples were obtained from them after 90 days since onset of illness (n =) to determine the persistence of NAbs. For detection of the NABs and their persistence, patients were recruited from day 14 to 21 (n = 98), day 22 to 28 (n = 100), day 29 to 36 (n = 132), day 37 to 42 (n = 32), day 43 to 49 (n = 16), day 50 to 70 (n = 29) and > 90 days (n = 76). The NAb titres were compared in those in whom we obtained a sample after 90 days of illness (n = 76), with the NAb during early illness (week 4 to 8 since onset of illness, time point A), and compared the titres to convalescence (> 90 days of illness, time point B).

### Determining the specificity of the sVNT

Sera from 81 patients who presented to the outpatient department of the NIID, in 2018 for treatment for a febrile illness were used to determine the specificity of the assay. Informed consent was obtained from patients with COVID-19 and those recruited from the outpatient department at NIID. 285 healthy individuals who were not exposed to individuals with COVID-19 were recruited from the Colombo Municipality Council area following informed written consent for further validation of the specificity of this assay.

### Ethical approval

Ethical approval was received by the Ethics Review Committee of Faculty of Medical Sciences, University of Sri Jayewardenepura. The study on humans were carried out in accordance with relevant guidelines and regulations (the Declaration of Helsinki).

### RT-PCR for detection of SARS CoV-2

Naso/Oro pharyngeal swabs or sputum samples of suspected SARS- CoV-2 patients were lysed and RNA was extracted using QIAmp Viral RNA Mini Kit (Qiagen, USA, Cat: 52,906) and used to detect the presence of N gene and ORF1ab gene of SARS-CoV2 with Da An Gene real time PCR kit (Da An Gene, China. Cat: DA-930) by real time RT PCR according to manufacturer’s instructions in ABI 7500 real time PCR system (Applied Biosystems, USA).

### Assay to measure NAb

As measuring SARS-CoV-2 NAbs would require a BSL-3 facility and limit the number of samples that can be assessed, we adopted a recently developed surrogate virus neutralization test (sVNT)^16^, which measures the percentage of inhibition of binding of the RBD of the S protein to recombinant ACE2 (Genscript Biotech, USA). Inhibition percentage ≥ 25% in a sample was considered as positive for NAbs.

### Statistical analysis

Data was analyzed by GraphPad Prism 8 version 8.4.2. The differences in NAb titres in patients at different time points with different disease severity was assessed using the two tailed Mann–Whitney U-test. The differences in NAb titres in those with mild/mild but prolonged shedding with those of severe or moderately severe illness was assessed again using the two tailed Mann–Whitney U-test. In order to determine the differences in the NAb titres in the same individual during early illness (week 4 to 8 since onset of illness, time point A), and convalescence (> 90 days of illness, time point B), we used the paired t test.
